# Supplementary material for: Systemic inflammation impairs microglial Aβ clearance through NLRP3 inflammasome
Source: EMBO J. 2019 Jul 30;38(17):e101064. doi: 10.15252/embj.2018101064 (PMC6717897; doi:10.15252/embj.2018101064)
Supplement: Supplementary file 1 — Appendix [file EMBJ-38-e101064-s001.pdf]

Table of contents:

Appendix Figure S1. Systemic inflammation triggers ASC speck formation. Page 1

Appendix Figure S2. Peripheral immune challenge affects amyloid deposition in hippocampus of APP/PS1 mice. Page 2

Appendix Figure S3. Amyloid plaque alters microglia morphology. Page 3

Appendix Figure S4. FACS analysis gating strategy. Page 4

Appendix Figure S5. No peripheral myeloid cell infiltration was observed in non-APP mice nor plaque-free areas upon LPS injection. Page 5

Appendix Figure S6. 3D automatic microglia reconstruction. Page 6

Appendix Figure S7. Two-photon imaging is performed in the same brain regions. Page 7

Appendix Figure S1

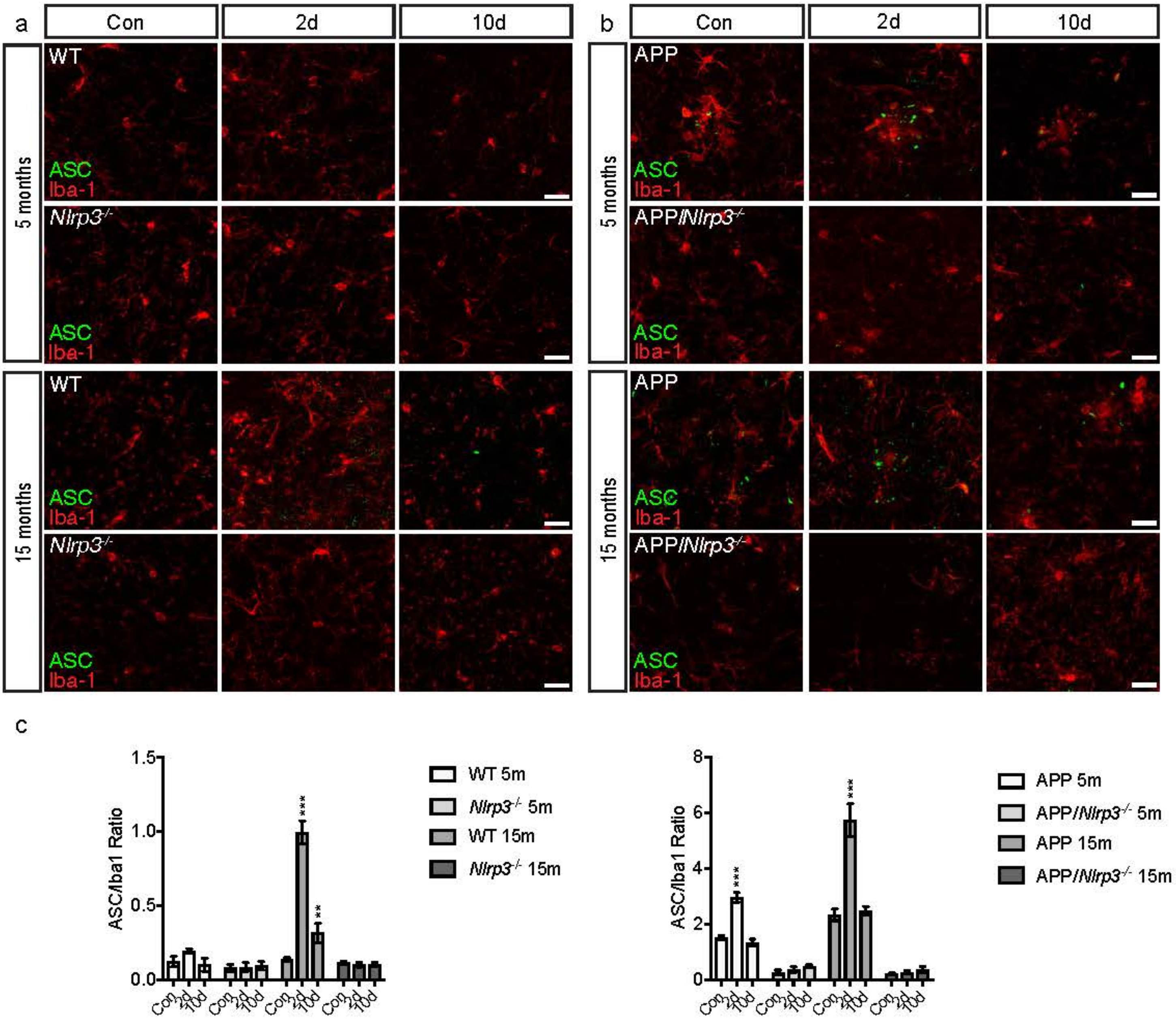

Appendix Figure S1. Systemic inflammation triggers ASC speck formation.
a) Iba-1 and ASC staining in 5 and 15 months old of wild-type and *Nlrp3*$^{-/-}$ mice. An increase in ASC specks formation (NLRP3-dependent) is observed 2 days after LPS injection. Scale bar: 20μm.
b) Iba-1 and ASC staining in 5 and 15 months old of APP and APP/*Nlrp3*$^{-/-}$ mice. Note the presence of ASC specks (NLRP3-dependent) in absence of peripheral immune challenge. LPS injection transiently increases the number of ASC specks in a NLPR3-dependent manner. Scale bar: 20μm.
c) Quantification of (a and b) respectively (mean of 5±SEM; two-way ANOVA followed Tukey's *post hoc* test, **$p<0.01$, ***$p<0.001$)

Appendix Figure S2

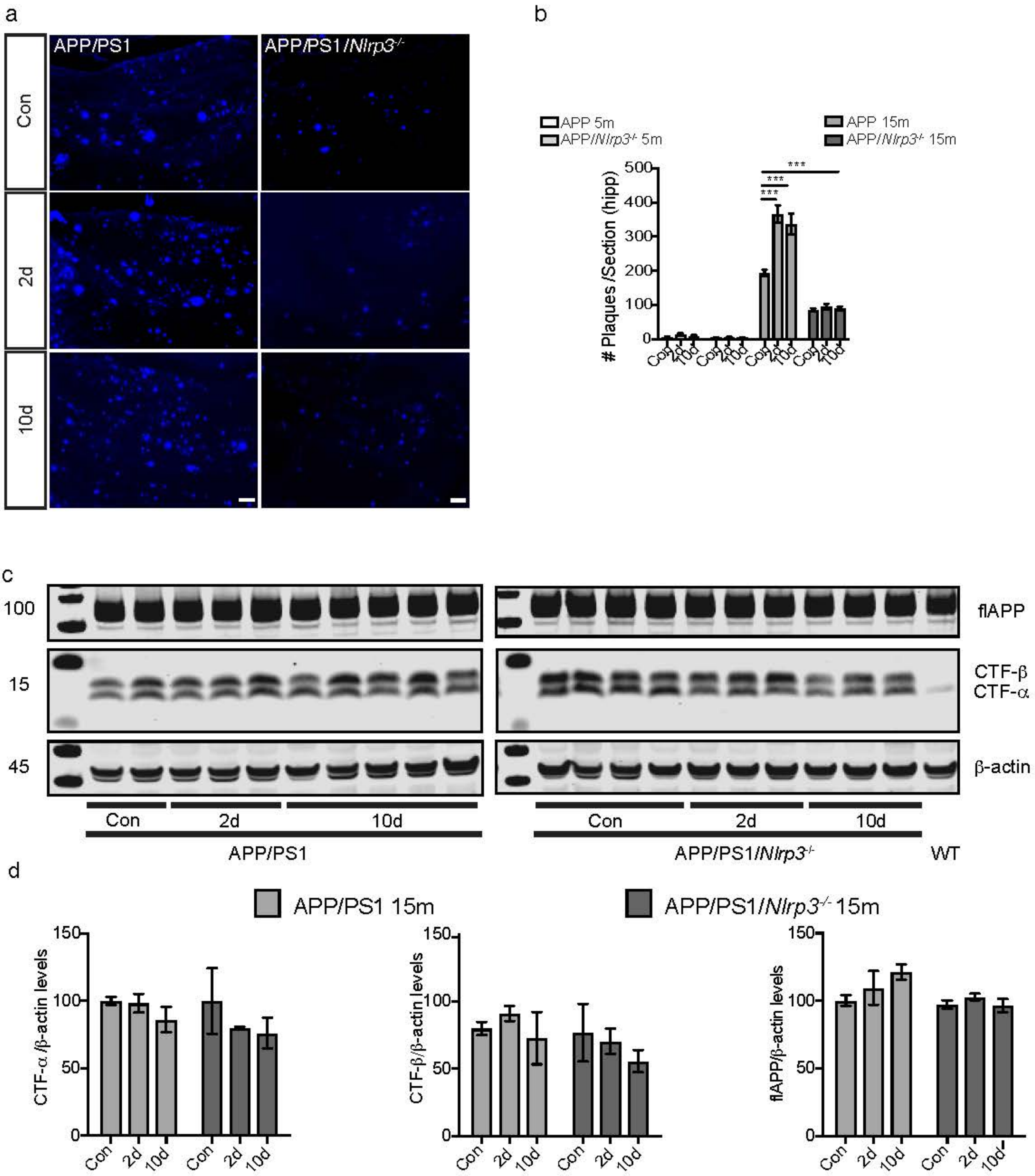

Appendix Figure S2. Peripheral immune challenge affects amyloid deposition in hippocampus of APP/PS1 mice.
a) Representative hippocampal images of MXO4 staining for APP and APP/*Nlrp3*$^{-/-}$ 15 months old mice. Scale bar: 50µm.
b) Hippocampal amyloid plaque number quantification, mean of 8±SEM; two-way ANOVA followed by Tukey's post hoc test, p ***$p<0.001$).
c) Western blot analysis of whole brain lysate from 15 months old APP and APP/*Nlrp3*$^{-/-}$ using CT20 antibody
d) Quantification of CTF-α, CTF-β and full length APP (c) expression (mean of 2-5±SEM)

Appendix Figure S3

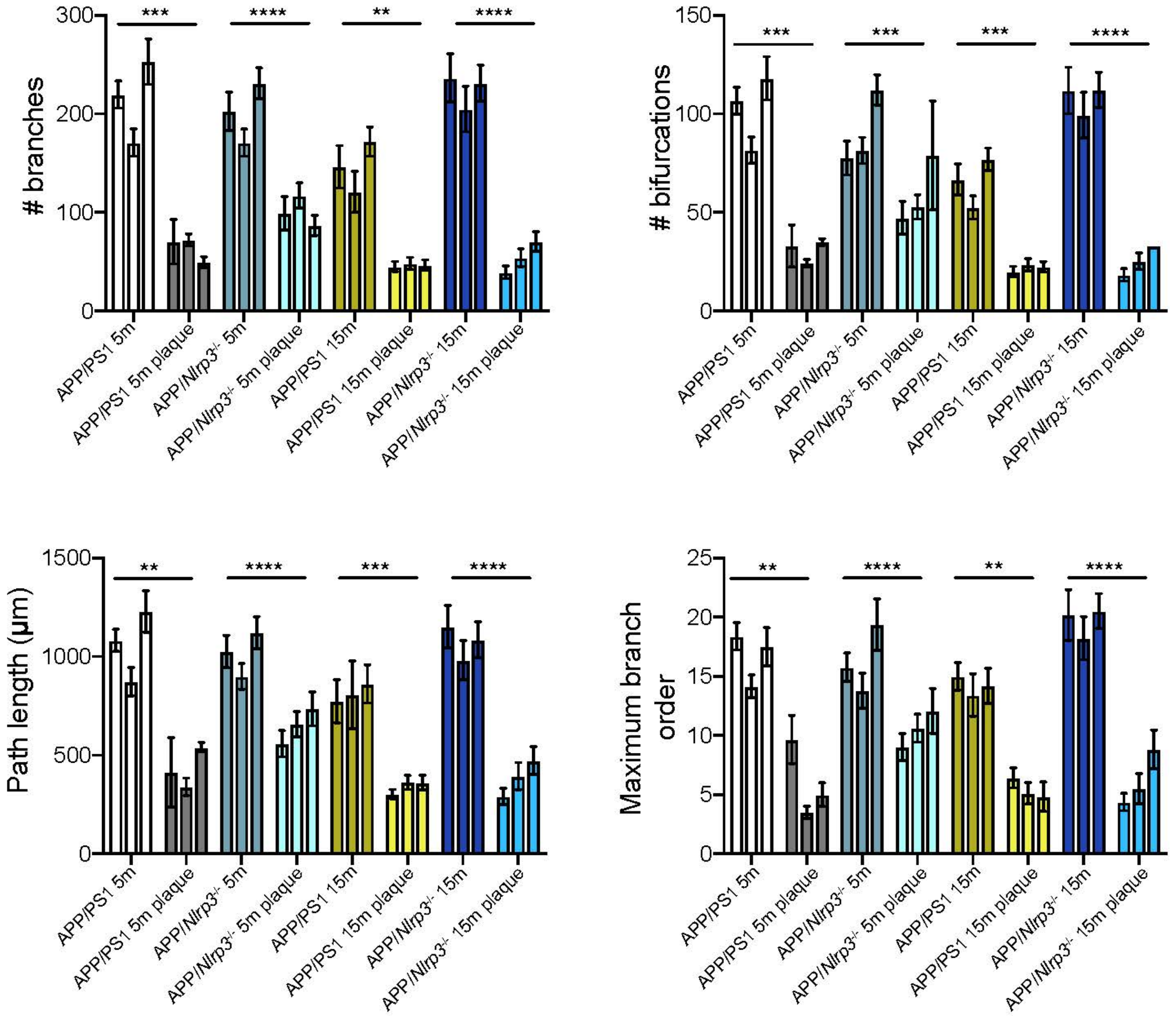

Appendix Figure S3. Amyloid plaque alters microglia morphology.
Quantification of microglia morphological parameters for APP and APP/*Nlrp3*$^{-/-}$ mice (mean of 5-6±SEM; two-way ANOVA followed by Tukey's post hoc test, **p<0.01, ***p<0.001).

Appendix Figure S4

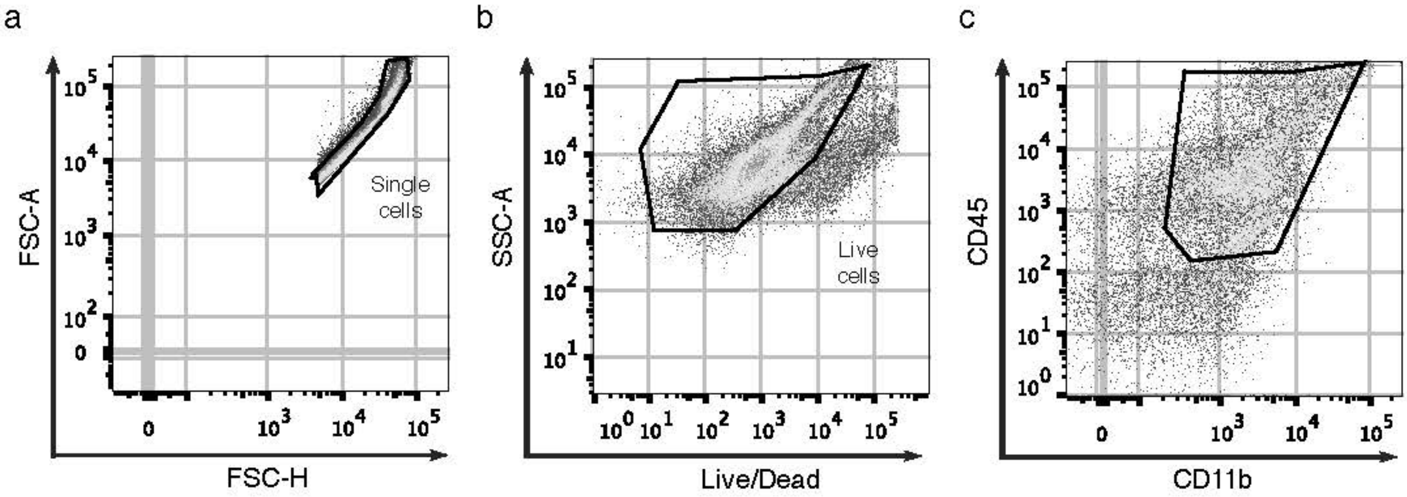

Appendix Figure S4. FACS analysis gating strategy.
a) Identification of individual cells.
b) Identification of live cells using live/dead staining.
c) Identification of microglia cells based on CD11b and CD45 staining

Appendix Figure S5

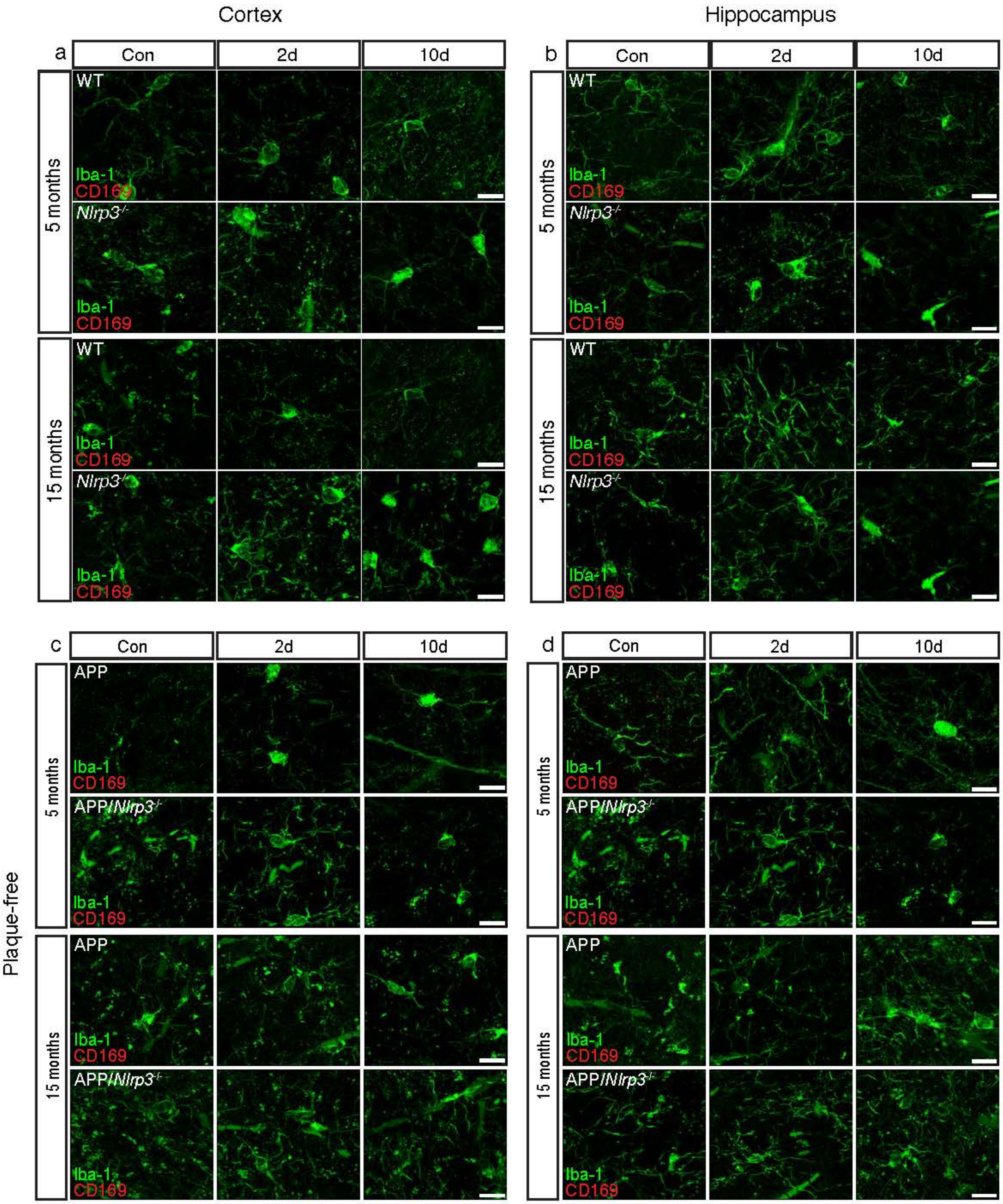

Appendix Figure S5. No peripheral myeloid cell infiltration was observed in non-APP mice nor plaque-free areas upon LPS injection.

a,b) Iba-1 (green) and CD169 (red) staining in cortex (left) and hippocampus (right) of 5 and 15 months old of wild-type and *Nlrp3*$^{-/-}$ mice. Scale bar: 20μm

c,d) Iba-1 (green) and CD169 (red) staining in plaque-free areas (cortex and hippocampus) of 5 and 15 months old of APP and APP/*Nlrp3*$^{-/-}$. Scale bar: 20μm

Appendix Figure S6

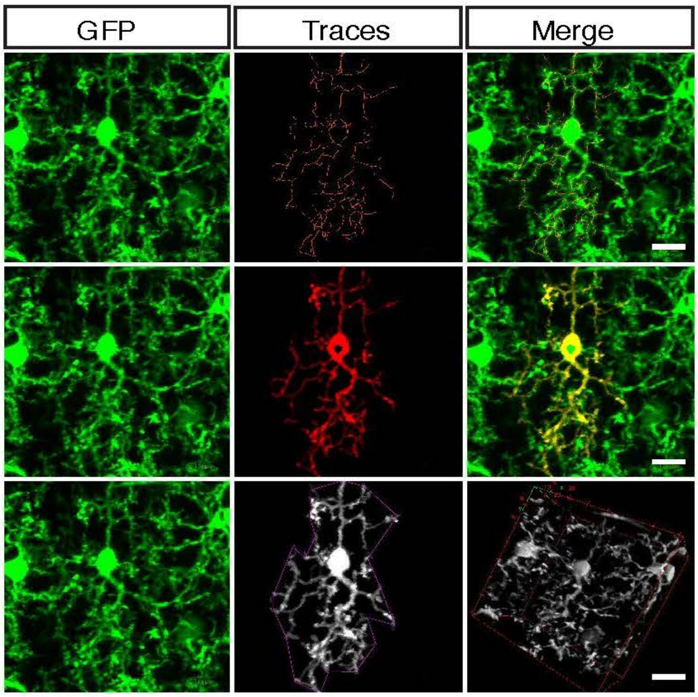

Appendix Figure S6. 3D automatic microglia reconstruction.
Example of in vivo 2-photon microglia reconstruction. Cx3cr1-eGFP microglia under 2-photon microscope (green) and its automatic traces reconstruction (red). Scale bar: 10μm.

Appendix Figure S7

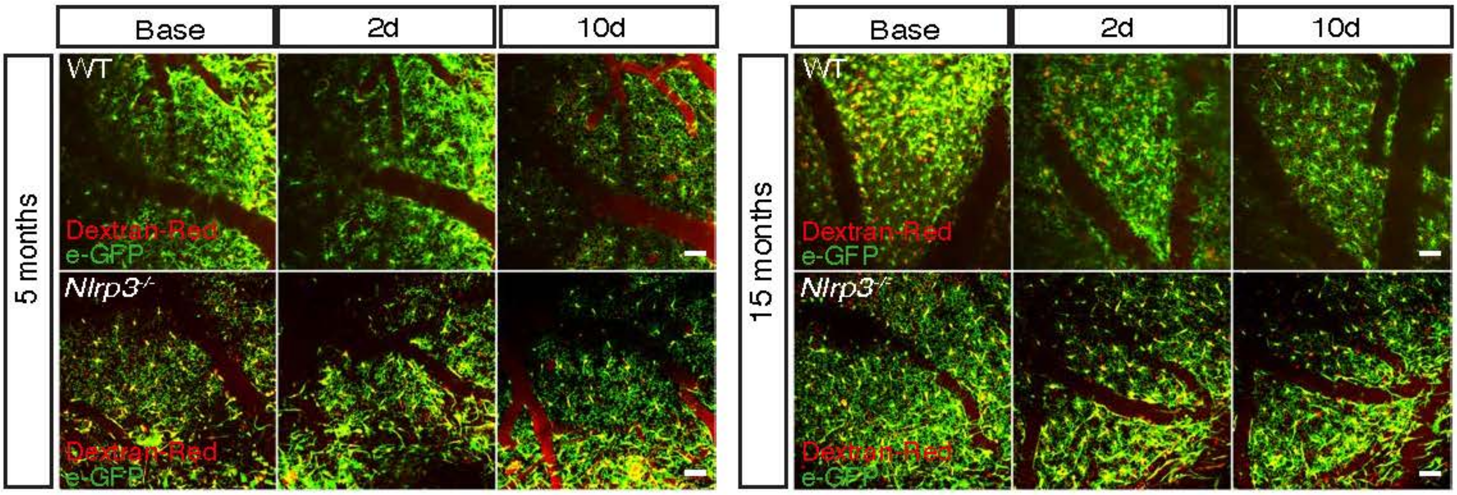

Appendix Figure S7. Two-photon imaging is performed in the same brain regions
Two-photon in vivo overview images from wild-type and *Nlrp3*$^{-/-}$ mice (5 and 15 months old). Imaging was performed on the very same brain regions across the time-points evidenced by vasculature (Dextran red). Scale bar: 50μm.
