# Supplementary material for: Systemic inflammation impairs microglial Aβ clearance through NLRP3 inflammasome
Source: EMBO J. 2019 Jul 30;38(17):e101064. doi: 10.15252/embj.2018101064 (PMC6717897; doi:10.15252/embj.2018101064)
Supplement: Supplementary file 2 — Expanded View Figures PDF [file EMBJ-38-e101064-s002.pdf]

Expanded View Figures

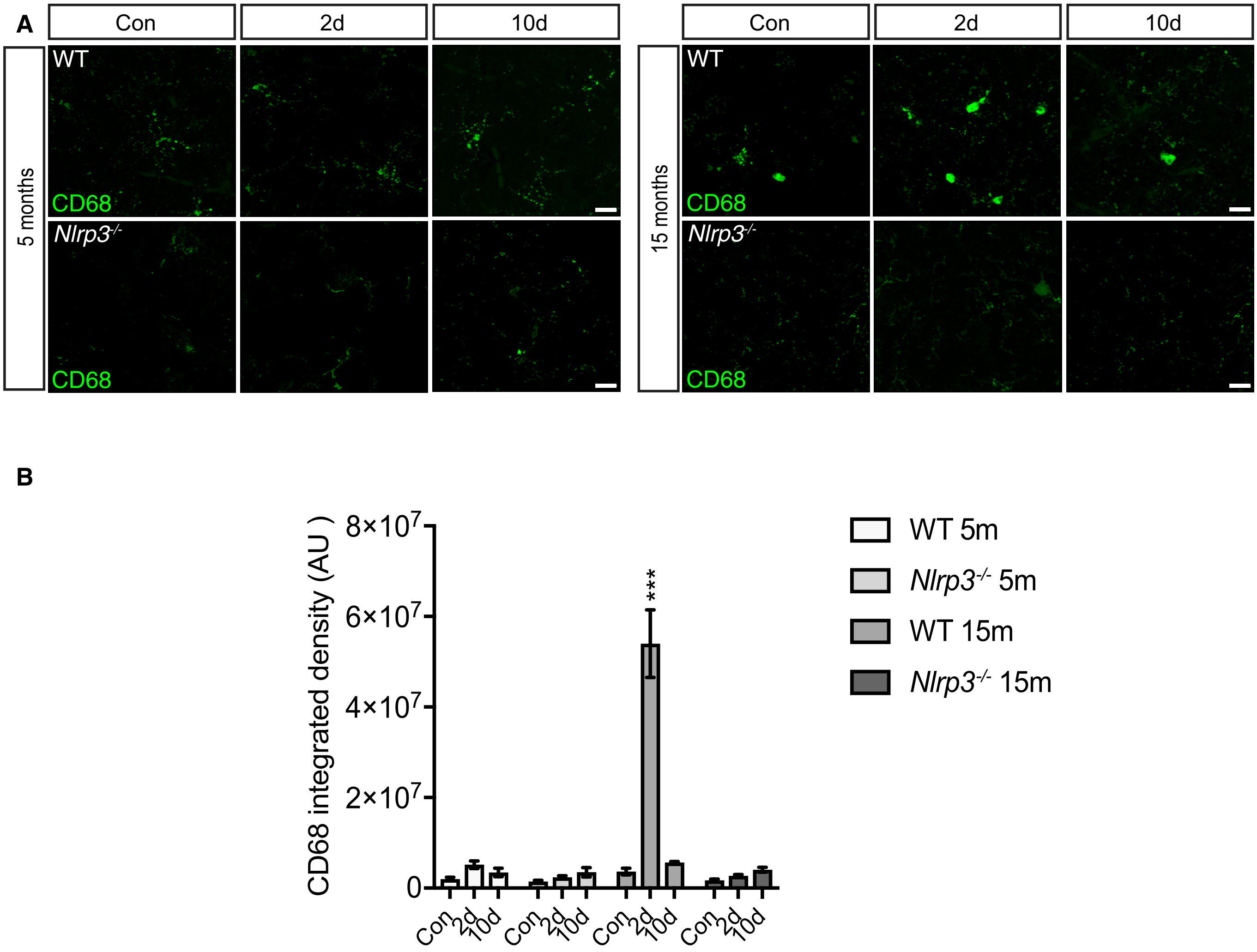

Figure EV1. Transient increase in CD68 immunoreactivity upon LPS injection.

A CD68 staining in cortex of 5 and 15 months old of wild-type and *Nlrp3*$^{-/-}$ mice. Scale bar: 20 μm.

B CD68 integrated density in wild-type and *Nlrp3*$^{-/-}$ mice (mean of 5 ± SEM; two-way ANOVA followed by Tukey's *post hoc* test, ***$P < 0.001$).

© 2019 The Authors

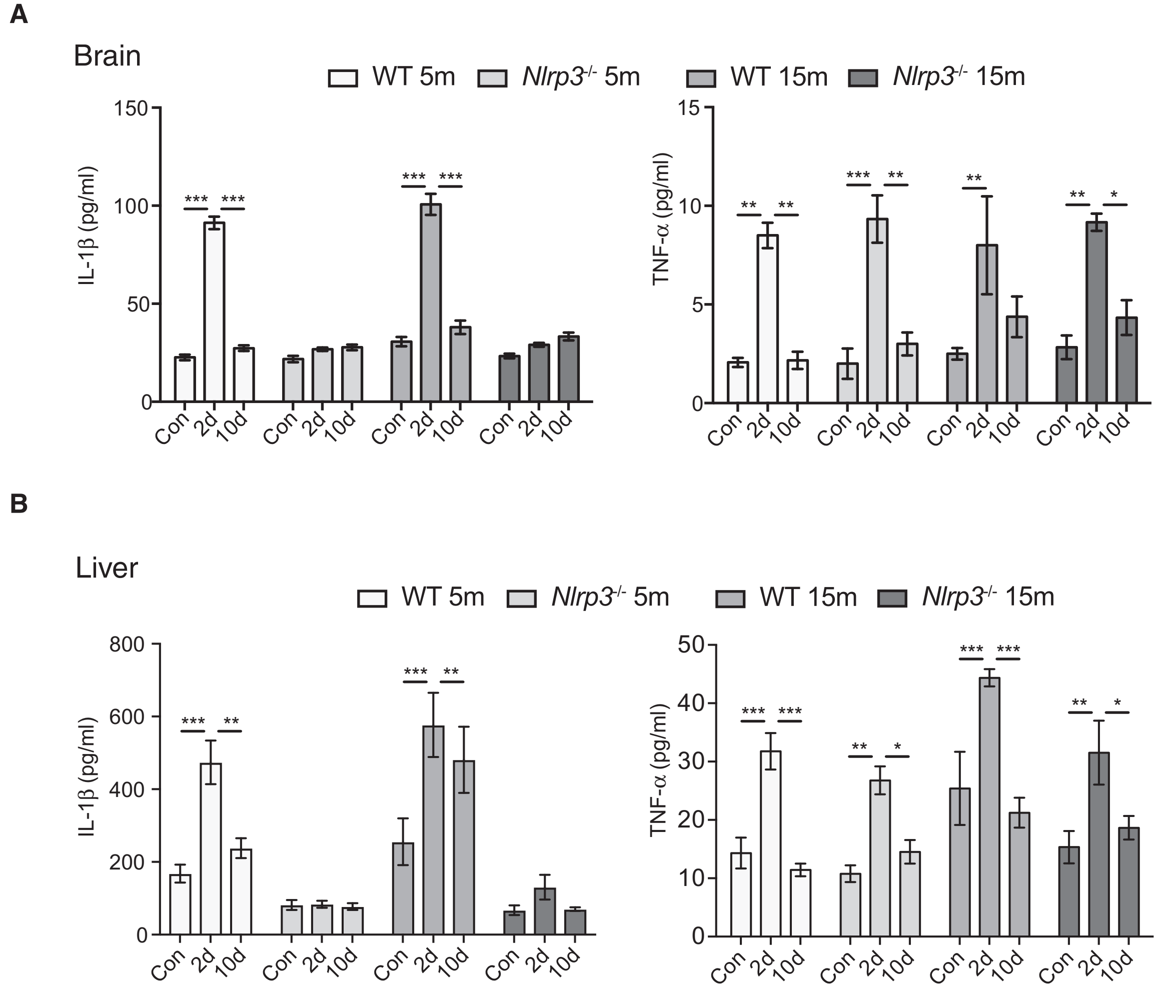

Figure EV2. LPS injection triggers a transient increase in pro-inflammatory cytokines in both brain and periphery.

A IL-1β and TNF-α ELISA measurement in brain lysates of wild-type and *Nlrp3*$^{-/-}$. A significant NLRP3-dependent increase of IL-1β levels was observed 2 days after LPS injection and then a return to control levels. For TNF-α, all groups showed a transient increase in its levels (mean of 6 ± SEM; two-way ANOVA followed by Tukey's *post hoc* test, $*P < 0.05$, $**P < 0.01$, $***P < 0.001$).

B IL-1β and TNF-α ELISA measurement in liver lysates of wild-type and *Nlrp3*$^{-/-}$ mice. A transient increase in NLRP3-dependent IL-1β levels is observed after immune challenge. LPS injection triggered a transient increase in TNF-α in all groups evaluated (mean of 6 ± SEM; two-way ANOVA followed by Tukey's *post hoc* test, $*P < 0.05$, $**P < 0.01$, $***P < 0.001$).

© 2019 The Authors

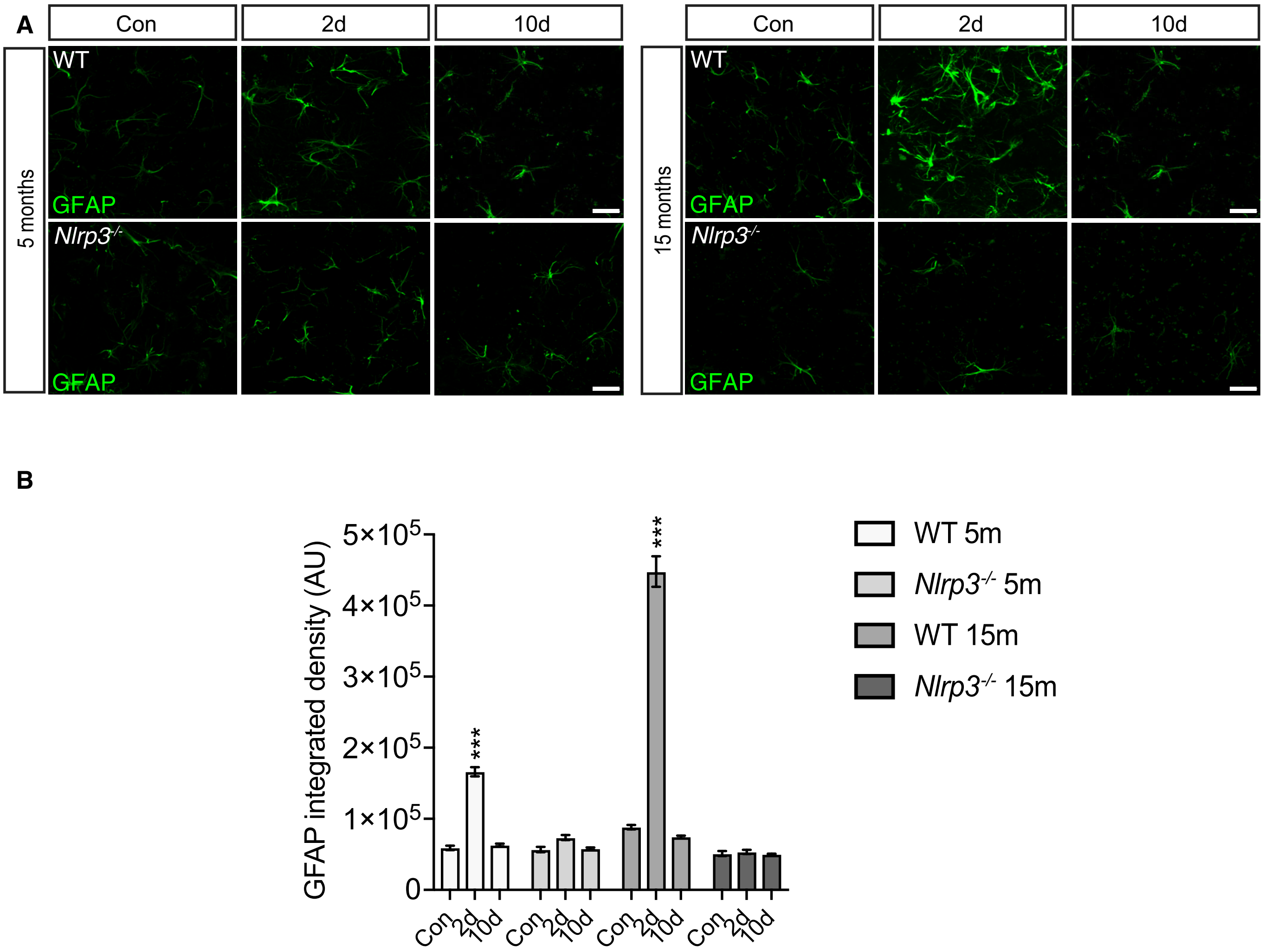

Figure EV3. Astrocytes are transiently activated after peripheral immune challenge.

A Representative cortical pictures of 5 and 15 months old wild-type and *Nlrp3*$^{-/-}$ stained with GFAP. A transient increase in GFAP immunoreactivity is observed 2 days after LPS injection in wild-type but not in *Nlrp3*$^{-/-}$ mice. Scale bar: 20 μm.

B GFAP integrated density in wild-type and *Nlrp3*$^{-/-}$ mice (mean of 5 ± SEM; two-way ANOVA followed by Tukey's *post hoc* test, ***$P < 0.001$).

© 2019 The Authors

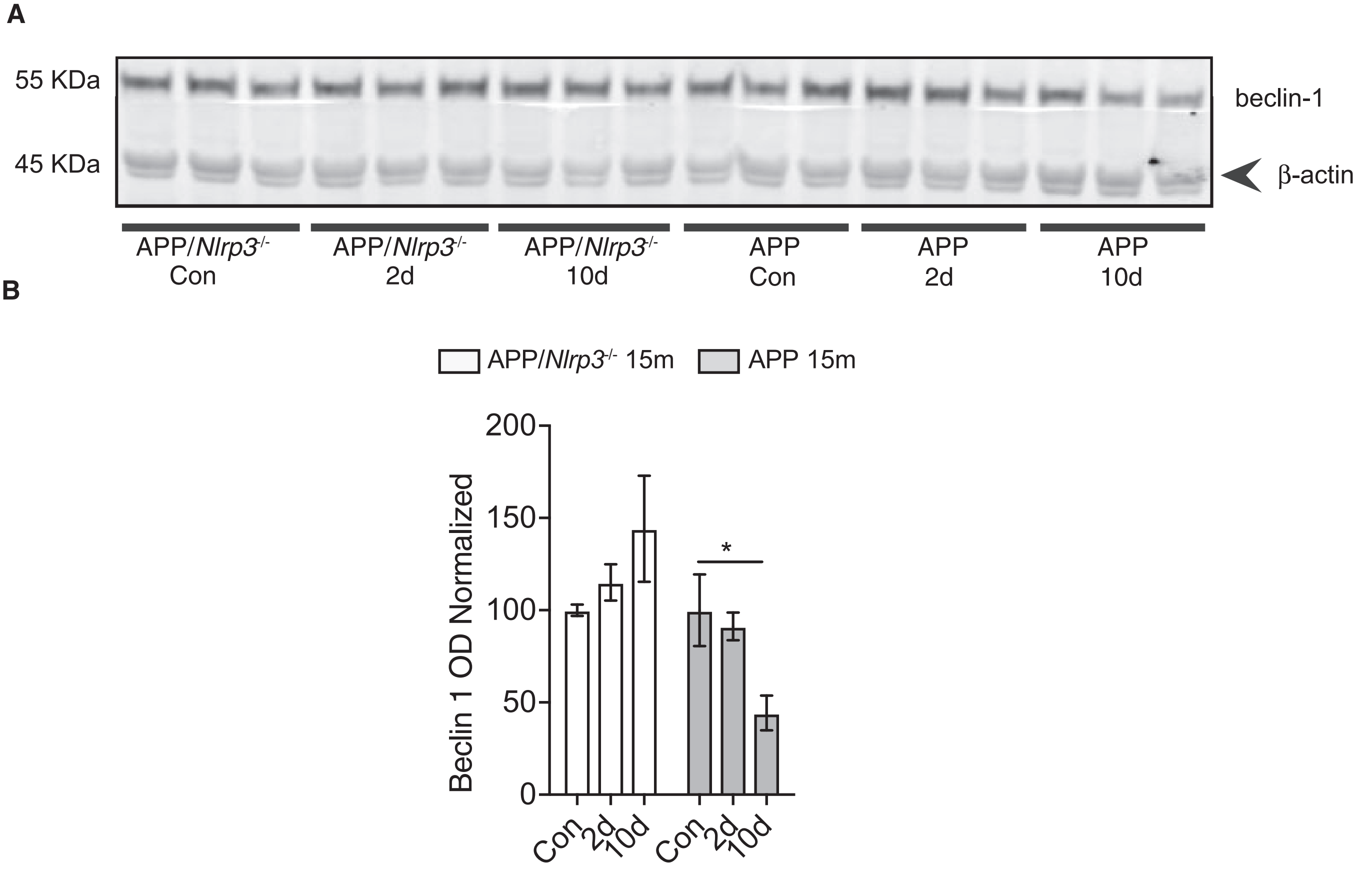

Figure EV4. Systemic inflammation alters beclin-1 expression in an NLRP3-dependent manner.

A Western blot analysis of whole brain lysate from 15-month-old APP and APP/*Nlrp3*$^{-/-}$ using beclin-1 antibody.

B Quantification of beclin-1 expression levels (mean of 2–5 ± SEM; two-way ANOVA followed by Tukey's *post hoc* test, *$P < 0.05$).

 © 2019 The Authors

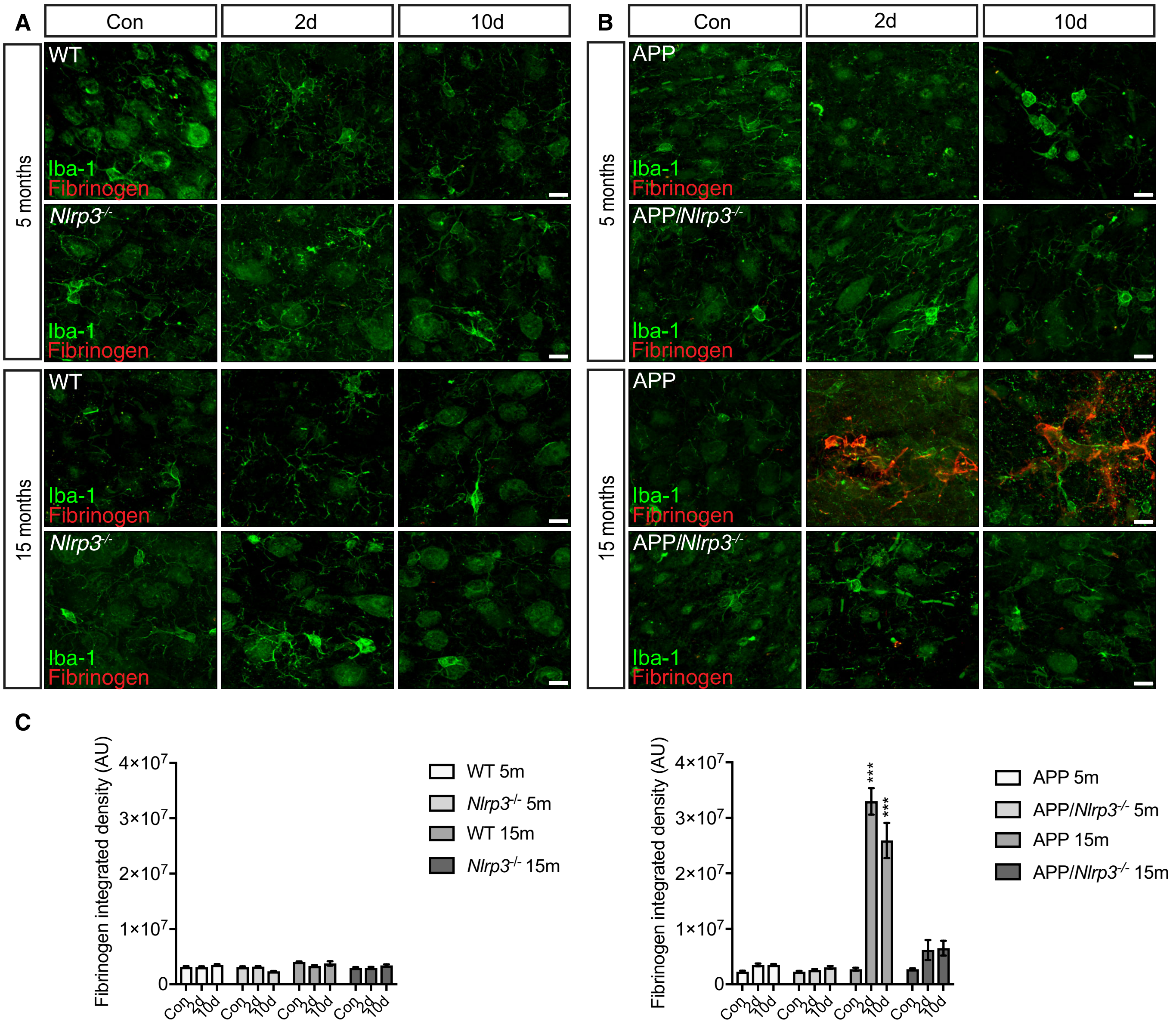

Figure EV5. Blood–brain barrier is compromised in old APP mice upon LPS injection.

A Iba-1 (green) and fibrinogen (red) staining in cortex of 5 and 15 months old of wild-type and *Nlrp3*$^{-/-}$ mice. Scale bar: 10 μm.

B Iba-1 (green) and fibrinogen (red) staining in cortex of 5 and 15 months old of APP and APP/*Nlrp3*$^{-/-}$ mice. Scale bar: 10 μm.

C Fibrinogen integrated density in wild-type and *Nlrp3*$^{-/-}$ mice (left panel) and APP and APP/*Nlrp3*$^{-/-}$ mice (right panel) (mean of 5 ± SEM; two-way ANOVA followed by Tukey's *post hoc* test, ****P* < 0.001).

© 2019 The Authors
